# A Deep Learning–Enabled Workflow to Estimate Real-World Progression-Free Survival in Patients With Metastatic Breast Cancer: Study Using Deidentified Electronic Health Records

**DOI:** 10.2196/64697

**Published:** 2025-05-15

**Authors:** Gowtham Varma, Rohit Kumar Yenukoti, Praveen Kumar M, Bandlamudi Sai Ashrit, K Purushotham, C Subash, Sunil Kumar Ravi, Verghese Kurien, Avinash Aman, Mithun Manoharan, Shashank Jaiswal, Akash Anand, Rakesh Barve, Viswanathan Thiagarajan, Patrick Lenehan, Scott A Soefje, Venky Soundararajan

**Affiliations:** 1 Department of Clinical Sciences, Nference, 4th Floor, Indiqube, Golf View Campus Tower-2, 22, 3rd Cross Rd, Murugeshpalya, S R Layout, Bangalore, 560017, India, 91 8728831787; 2 Department of Data Science and Engineering, Nference, Bangalore, India; 3 Department of Clinical Sciences, Nference, Cambridge, MA, United States; 4 Department of Pharmacy, Director of Pharmacy, Mayo Clinic, Rochester, MN, United States

**Keywords:** real-world evidence, data-driven oncology, real-world progression-free survival, metastatic breast cancer, natural language processing, NLP, survival, cancer, oncology, breast, metastatic, deep learning, machine learning, ML, workflow, report, notes, electronic health record, EHR, documentation

## Abstract

**Background:**

Progression-free survival (PFS) is a crucial endpoint in cancer drug research. Clinician-confirmed cancer progression, namely real-world PFS (rwPFS) in unstructured text (ie, clinical notes), serves as a reasonable surrogate for real-world indicators in ascertaining progression endpoints. Response evaluation criteria in solid tumors (RECIST) is traditionally used in clinical trials using serial imaging evaluations but is impractical when working with real-world data. Manual abstraction of clinical progression from unstructured notes remains the gold standard. However, this process is a resource-intensive, time-consuming process. Natural language processing (NLP), a subdomain of machine learning, has shown promise in accelerating the extraction of tumor progression from real-world data in recent years.

**Objectives:**

We aim to configure a pretrained, general-purpose health care NLP framework to transform free-text clinical notes and radiology reports into structured progression events for studying rwPFS on metastatic breast cancer (mBC) cohorts.

**Methods:**

This study developed and validated a novel semiautomated workflow to estimate rwPFS in patients with mBC using deidentified electronic health record data from the Nference nSights platform. The developed workflow was validated in a cohort of 316 patients with hormone receptor–positive, human epidermal growth factor receptor-2 (HER-2) 2-negative mBC, who were started on palbociclib and letrozole combination therapy between January 2015 and December 2021. Ground-truth datasets were curated to evaluate the workflow’s performance at both the sentence and patient levels. NLP-captured progression or a change in therapy line were considered outcome events, while death, loss to follow-up, and end of the study period were considered censoring events for rwPFS computation. Peak reduction and cumulative decline in Patient Health Questionnaire-8 (PHQ-8) scores were analyzed in the progressed and nonprogressed patient subgroups.

**Results:**

The configured clinical NLP engine achieved a sentence-level progression capture accuracy of 98.2%. At the patient level, initial progression was captured within ±30 days with 88% accuracy. The median rwPFS for the study cohort (N=316) was 20 (95% CI 18-25) months. In a validation subset (n=100), rwPFS determined by manual curation was 25 (95% CI 15-35) months, closely aligning with the computational workflow’s 22 (95% CI 15-35) months. A subanalysis revealed rwPFS estimates of 30 (95% CI 24-39) months from radiology reports and 23 (95% CI 19-28) months from clinical notes, highlighting the importance of integrating multiple note sources. External validation also demonstrated high accuracy (92.5% sentence level; 90.2% patient level). Sensitivity analysis revealed stable rwPFS estimates across varying levels of missing source data and event definitions. Peak reduction in PHQ-8 scores during the study period highlighted significant associations between patient-reported outcomes and disease progression.

**Conclusions:**

This workflow enables rapid and reliable determination of rwPFS in patients with mBC receiving combination therapy. Further validation across more diverse external datasets and other cancer types is needed to ensure broader applicability and generalizability.

## Introduction

### Background and Significance

Real-world evidence (RWE) is increasingly accepted to augment traditional clinical trial findings to better understand the effectiveness of oncological interventions. RWE can be leveraged to improve novel therapy development programs and provide better postmarket surveillance of approved therapies [1-3].

Response evaluation criteria in solid tumors (RECIST) is broadly used to ascertain disease progression in clinical trials. However, assessing RECIST in retrospective electronic health record (EHR) data is challenging due to its strict assessment indicators [4]. RECIST considers changes in the size of individual target lesions over time and the presence or absence of new lesions to categorize disease status into complete or partial response, stable disease, or progression [5]. A similar assessment paradigm is adopted in routine clinical practice, where clinicians document the occurrence of progression through serial clinical and radiological examinations. This clinician-confirmed cancer progression in unstructured text (ie, clinical notes) has been shown to serve as a reasonable surrogate for real-world indicators in ascertaining progression endpoints. This is also more practical for real-world studies than purely RECIST-based approaches [6].

In earlier studies across different types of solid tumors, real-world progression (rwP) was captured either through manual abstraction from unstructured data or proxy measures were evaluated based solely on structured drug data [7,8]. Recent studies have also used machine learning models specifically trained to automate the capture and characterization of clinician documentation of tumor response. These specialized models have shown varying accuracies [9,10]. In the past decade, health care natural language processing (NLP) frameworks like Google’s Healthcare Natural Language application programming interface (API), Amazon Comprehend Medical, IBM Watson Health, and Microsoft Text Analytics for Health have emerged and shown promise in clinical concept recognition, entity linking, and sentiment analysis. However, these general-purpose NLP frameworks have shown varying degrees of performance on different data sources [11-13]. While large language models (LLMs) are rapidly advancing, they currently have limitations in clinical concept identification and medical relation extraction as structured outputs for direct application. Even specialized clinical LLMs require further fine-tuning for such use cases [14].

We aim to configure a pretrained, general-purpose health care NLP framework to transform free-text clinical notes and radiology reports into structured progression events. By combining these with structured drug records and encounter data, we will compute real-world progression-free survival (rwPFS) for metastatic breast cancer (mBC). This work can also guide other researchers in configuring a general-purpose health care NLP model to capture rwPFS. Developing a standardized and automated path for ascertaining rwP could help scale rwPFS computation across diverse subsets of solid tumors and maintain a better agreement across real-world studies.

### Objectives

We aim to (1) develop and validate a novel semiautomated workflow that estimates rwPFS in patients with mBC, (2) explore the essentiality of each model in the general-purpose NLP framework through ablation analysis, and (3) investigate additional factors influencing rwPFS, such as the source of clinician-documented progressions (radiology reports versus routine clinical notes) and the presence of prior or concurrent medications during the observation period.

## Method

### Data Source

This study analyzed deidentified EHR data from a network of tertiary clinical centers tied to an academic medical center (Mayo Clinic) in the United States through the Nference nSights Analytics Platform [15]. In-house tools were used for the deidentification of EHRs. The tool performs with a recall of 0.992 and a precision of 0.979 on the i2b2 2014 dataset at replacing protected health information (PHI) with plausible but fictional surrogates [16]. Overall, the platform hosts data from approximately 7 million patients from across the United States of America, with about 1.8 million patients having a mention of cancer across the structured data. The platform hosts an array of multimodal data, both structured and unstructured, such as clinical notes, radiology reports, Digital Imaging and Communications in Medicine headers, and pathology reports.

### Study Design and Definitions

**Figure 1.**
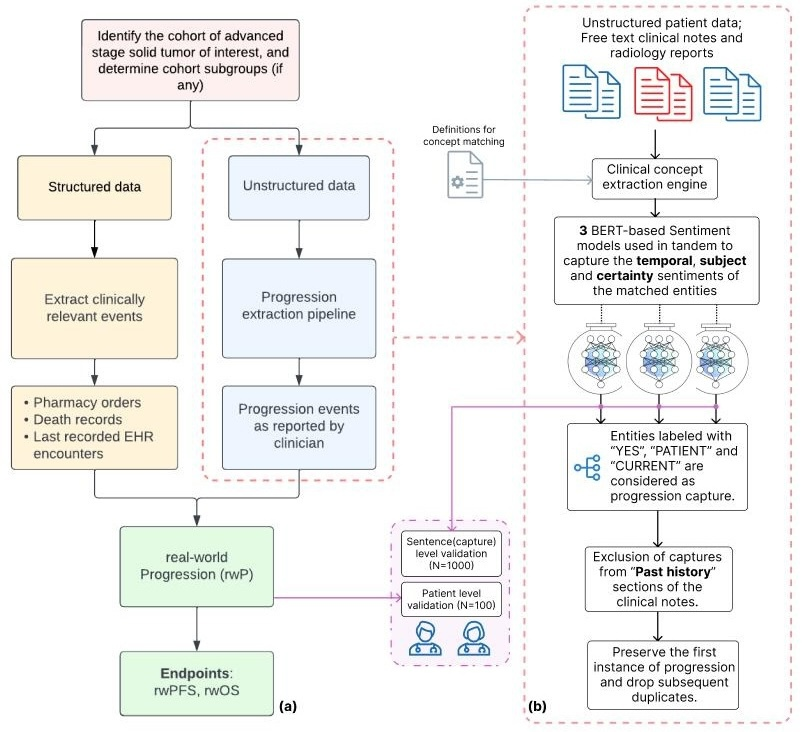
Methodology flow diagram illustrating the workflow. (**A**) Workflow for real-world progression (rwP) extraction and determining the real-world progression-free survival (rwPFS). (**B**) The methodology for capturing progression from unstructured texts in routine clinical documents and radiology reports using Nference’s clinical NLP engine that performs clinical concept recognition, association, and sentiment analysis. BERT: Bidirectional Encoder Representations from Transformers; EHR: electronic health record.

This retrospective observational study demonstrates the estimation of rwPFS with a workflow that integrates clinician-reported progression events from free text (unstructured data) in clinical and radiology documents with structured patient events like drug orders, clinic or hospital encounters, and mortality records. The workflow was developed to leverage the pretrained, general-purpose, deep learning–based health care NLP framework developed at Nference called the clinical NLP engine, which enables clinical concept recognition, sentiment analysis, and linking associated concepts. The models that are a part of the clinical NLP engine were initially trained on human-annotated datasets, and later further augmented by additional ground truth datasets generated by LLM agents from the same parent EHR data source. A high-level overview of the workflow is illustrated in Figure 1.

### Data Extraction and Augmentation

**Figure 2.**
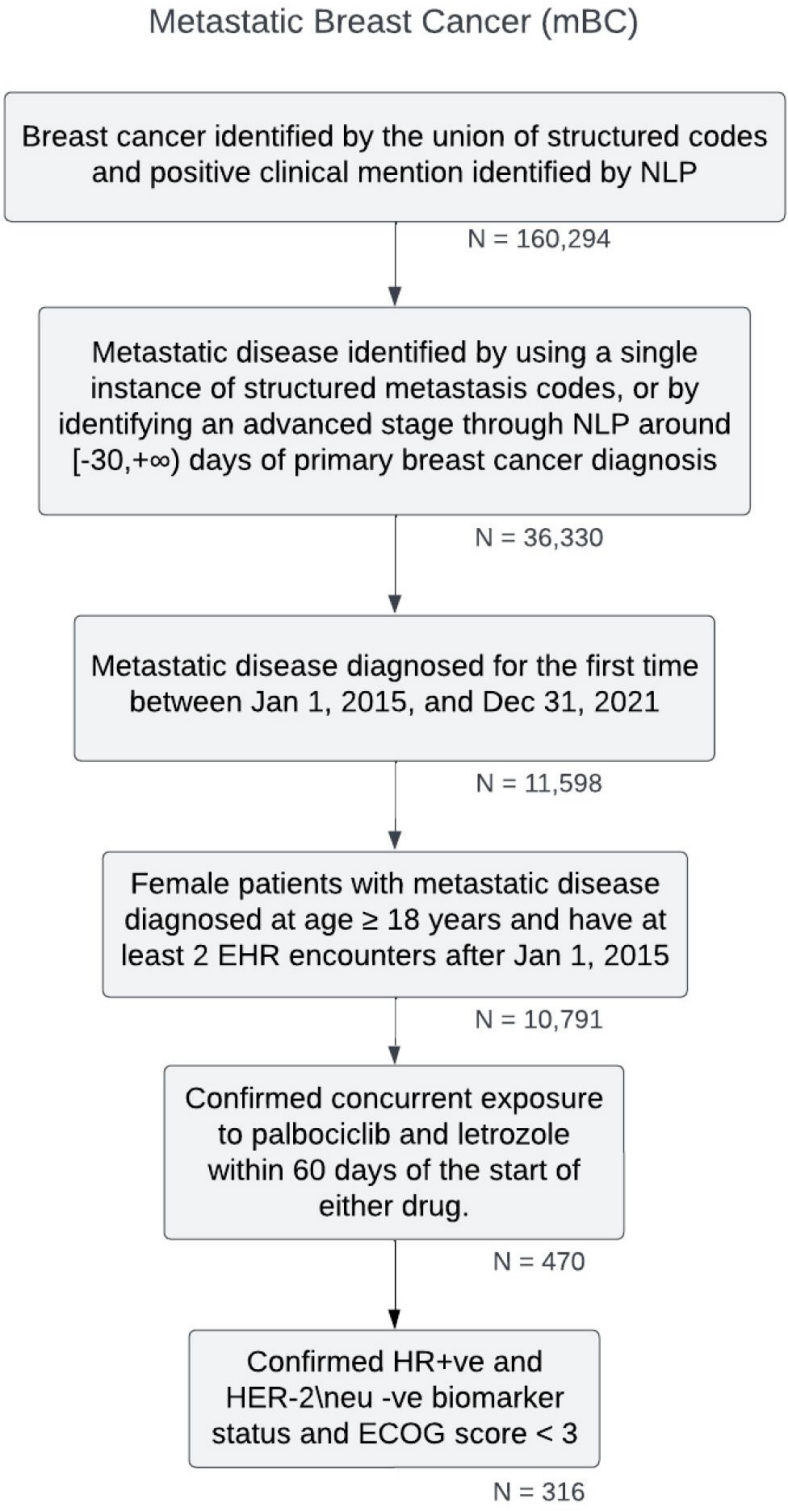
Cohort attrition diagram: structured codes 174* (*ICD-9*) and C50* (*ICD-10*) or >4 positive disease sentiments from the augmented curation disease diagnosis model were used for breast cancer. For evidence of metastasis, 197*, 198* (*ICD-9*), C78*, and C79* (*ICD-10*) in conjunction with augmented curation were used; * represents all the children codes within the parent code. ECOG: Eastern Cooperative Oncology Group; EHR: electronic health record; HER-2: human epidermal growth factor receptor-2; HR: hormone receptor; *ICD-9*: *International Classification of Diseases, Ninth Revision*; *ICD-10*: *International Statistical Classification of Diseases, Tenth Revision*; NLP: natural language processing.

Breast cancer disease was identified using structured diagnosis codes 174 (*ICD-9* [*International Classification of Diseases, Ninth Revision*]), C50 (*ICD-10* [*International Statistical Classification of Diseases, Tenth Revision*]), and NLP-based positive model confirmations (augmented curation) of the disease-related terms in clinical notes [17]. A similar approach was undertaken for identifying metastatic disease using structured codes 197, 198 (*ICD-9*), C78, and C79 (*ICD-10*). Further cohort attrition for the study population is outlined in Figure 2. Hormone receptor status, human epidermal growth factor receptor-2 (HER-2) status, and Eastern Cooperative Oncology Group scores were captured from clinical notes using the clinical NLP engine. A rule-based approach was used to identify the initiation date of first-line therapy in mBC by analyzing drug orders and administration records. To ensure reliability, only orders appearing for the first time after metastasis diagnosis were included. The same methodology was extended to identify second-line therapies.

### Patient Population

The study cohort (N=316) consisted of female patients aged ≥18 years and diagnosed with mBC with hormone receptor–positive and HER-2–negative status with confirmed concurrent exposure to palbociclib and letrozole from January 1, 2015, to December 31, 2021 (study period), and with Eastern Cooperative Oncology Group performance scores of less than 3 around the start of the therapy (±60 d).

Demographics and baseline characterization of the study cohort, such as prior exposure to therapies, disease stage at the start of the study, stage at first cancer diagnosis, and other relevant metrics for the solid tumor of interest, were also documented.

### Extracting Progression Events From Unstructured Text

To develop and evaluate our workflow, an initial rule-definition set of 200 cases from the overall mBC cohort (N=10,791) was sampled, and a preliminary manual abstraction was performed. This evaluation aimed to systematically identify a set of rules for configuring the baseline clinical concept extraction model to capture progression. Authors PKM, SKR, VK, and MM used internal clinical document (CD) exploration tools to cluster sentences based on initial pattern matches and iteratively refined these clusters to identify progression-indicative phrases. Independent reviews of oncology and radiology notes were conducted to extract commonly occurring phrases indicative of clinical progression. Authors GV and RHY subsequently collated these identified patterns into a set of regex search patterns. These patterns were tested on clinical notes to ensure they captured the appropriate progression-related contexts while removing duplicate or irrelevant verbatim such as general report headers, unrelated phrases (eg, “CR” for complete response or “PD” for progressive disease without patient-specific context), and noise. Downstream NLP models were applied to validate the extracted patterns by mapping the right set of label combinations that accurately reflected the progression status. This process was repeated iteratively until a consensus was reached among the authors, ensuring a robust set of rules for capturing progression events. The final progression capture configuration is detailed in Table S1 in Multimedia Appendix 1.

The rwP events were captured by configuring the clinical NLP engine. This baseline workflow is an ensemble of deep learning–based multi-BERT (Bidirectional Encoder Representations from Transformers) framework trained on unstructured patient data like CDs and radiology reports to perform named entity recognition and predict the sentiment labels for *subject*, *temporality*, and *certainty* of the captured named entities [18]. The ensemble also infers associations between related entities like disease-severity, drug-disease, and disease-anatomical_structure, among others. These proprietary models are fine-tuned versions of SciBERT-cased [19], a domain-specific transformer model pretrained on scientific text. The base models underwent further supervised fine-tuning for classification tasks on annotated sentences from CD texts of the overall Nference nSights database, but not specifically on the mBC patient note database. The details regarding their architecture, training, and performance of the individual models of the clinical NLP engine are detailed in Note S1 and Table S2 in Multimedia Appendix 1. The clinical NLP engine also uses a section header model to identify the clinical note sections from which the named entities were captured. The rules determined during the initial abstraction were used to capture cancer progression.

### rwP Definitions

rwP events were identified by the earliest documentation of disease progression in a clinical or radiology note or by advancement to a new line of therapy. The addition of a new chemotherapy, endocrine therapy, or targeted therapy drug after 60 days of exposure to the initiating therapy of interest is considered line advancement. The following list of drugs were considered potential second-line drug candidates in the study period: tamoxifen, fulvestrant, elacestrant, paclitaxel, carboplatin, abemaciclib, docetaxel, cyclophosphamide, capecitabine, ribociclib, alpelisib, everolimus, doxorubicin, epirubicin, 5-fluorouracil, olaparib, talazoparib, ixabepilone, raloxifene, and toremifene. Censoring events included death, loss of follow-up, and the end of the observation period. Progression events captured within the first 30 days of therapy initiation were excluded as they occurred too early to reflect treatment effectiveness. The rwPFS was also assessed with variations in the origin of unstructured data, comparing radiology reports and CDs as data sources. The key contributing survival variables used for rwPFS were also stratified to understand the source of events.

### Progression Capture Validation

#### Overview

For validation of the clinical progression captures, the manual review and abstraction were performed at 2 levels.

The raw progression captures were evaluated for accuracy independent of their temporality to the observation period. 1000 captures were sampled from the overall pool of progression captures for the sentence-level progression capture analysis.

A stratified sample (mBC validation set; n=100) was selected from the overall study cohort (N=316) to match the progression event occurrence observed in the overall set. This approach ensures that the sample mirrors the broader cohort’s characteristics for a valid comparison in patient-level evaluation for progression capture. These patients were not part of the initial rule-definition set and were evaluated for their first progression events. For the patient-level analysis, the elements of the confusion matrix were defined to account for temporality. We classified the captures into 4 categories: (1) true positives: automated progression captured is within ±30 days of manual capture; (2) false positives: progression was not found through manual capture, but automated progression was captured at any time or automated progression was captured >30 days before manual capture; (3) true negatives: progression was not found through manual review, nor was picked up by automated capture; and (4) false negatives: progression was identified through manual capture, but the automated method has not identified any progression (or) automated method captured progression >30 days after the date captured by manual review.

#### Ablation Analysis of the Progression Capture Pipeline

To evaluate the contribution of each workflow component to the overall performance, an ablation study was performed at 5 strategic points: (1) temporal model ablation, that is, the removal of the temporality assessment model; (2) subject model ablation, that is, the removal of the subject assessment model; (3) certainty model ablation, that is, the certainty assessment model was removed; (4) all 3 assessment models were removed; and (5) postprocessing ablation where the postprocessing steps, specifically the exclusion of specific note sections and dropping subsequent duplicate mentions of the same note contexts, were removed. Each ablation was analyzed in isolation to quantify its respective contribution to the final output’s accuracy, aiding in identifying critical components of the pipeline and potential areas for optimization. This step is further illustrated in Figure S2 in Multimedia Appendix 1.

### Validation on the External Dataset

To further assess the generalizability and robustness of the progression capture pipeline, external validation was performed using data from a different partner academic medical center (AMC). In the external dataset, 63 mBC patients on first-line therapy of the metastatic disease with palbociclib with or without aromatase inhibitors were identified for this analysis (see Figure S4 for cohort attrition in Multimedia Appendix 1). Similar NLP-based data augmentation techniques were applied on the external dataset for cohort identification. The proposed progression extraction workflow was applied on the external dataset for extraction of progression events during the defined study period (60 months) of the patients. For validation, similar to the primary cohort, a 2-tier approach was applied, including sentence-level and patient-level validation. For patient-level validation, the time of the first progression event in the patient cohort was manually abstracted and validated. Performance metrics such as precision, recall, accuracy, and *F*_1_-score were calculated to evaluate the alignment of automated progression captures with manual annotations.

### Sensitivity Analysis on rwPFS Estimates

Two sensitivity analyses were conducted to assess the robustness of rwPFS estimates: (1) to evaluate the effect of data incompleteness, 10%, 20%, and 30% of rows capturing progression events (missingness at random) were systematically removed from the Kaplan-Meier source data. Median rwPFS and survival probabilities were descriptively analyzed to quantify variations introduced by missing records. (2) The impact of treating death as a progression event versus censoring was assessed by generating Kaplan-Meier curves under both scenarios. Differences were evaluated using the log-rank test, with comparisons of median rwPFS and survival probabilities at predefined time points. These analyses ensured the robustness of rwPFS estimates by addressing potential biases from data structure and event definitions.

### Patient-Reported Outcomes and Clinical Progression: Analysis Using the Patient Health Questionnaire-8

Patient-reported outcomes (PROs) were integrated by analyzing Patient Health Questionnaire-8 (PHQ-8) scores to complement clinician-documented progression events. Cumulative declines and peak reductions in PHQ-8 scores were compared between progressed and nonprogressed patients using a *t* test. Peak reduction was determined as the largest decrease observed between any 2 recorded scores during the study period. Cumulative decline, representing the total improvement over time, was calculated as the sum of all positive reductions (decline in PHQ-8 value) in scores across all pairwise comparisons during the study period. This approach aimed to provide a holistic perspective by linking patient-reported mental health outcomes to clinical progression.

### Outcomes Assessment

The primary outcome was rwPFS, calculated as the time between the start of the intervention of interest and the first rwP event captured or a change in the line of therapy for the patient. The secondary outcome was real-world overall survival (rwOS), calculated as the time between the start of the intervention of interest and the date of death. A subgroup analysis was performed to assess the impact of prior and other concomitant medications on rwPFS and rwOS in the metastatic setting. The validation metrics of the primary outcome were reported as sensitivity, specificity, accuracy, precision, and *F*_1_-scores.

Median follow-up time was computed from the date of the start of therapy till their last encounter in the EHR system. Time to treatment after the diagnosis of advanced disease (first evidence of metastasis) and follow-up after the start of therapy were imputed using *the date of the first evidence of metastasis* (identified by structured disease code or NLP-positive confirmation) and *the date of the first structured drug order for the combination therapy* (palbociclib and letrozole) as the anchor dates, respectively.

### Ethical Considerations

This study analyzed deidentified primary patient-level data extracted from the Nference’s, nSights electronic health record database under a data-use agreement that obviates the need for additional institutional review board review. Nference, in collaboration with the AMC data partner that provided the deidentified data for this study, has established a secure data environment, hosted by and within the AMC, that houses the AMC’s deidentified patient data. The provisioning of and access to this data are governed by an expert determination that satisfies the Health Insurance Portability and Accountability Act Privacy Rule requirements for the deidentification of PHI. Each AMC’s deidentified data environment is specifically designed and operated to enable access to and analysis of deidentified data without the need for institutional review board oversight, approval, or an exemption confirmation. Participants retain the right to opt out at any time. The data are accessible only to authorized users subject to a robust credentialing and authentication process. Data shown and reported in the manuscript have been extracted from this environment using an established protocol for data extraction, aimed at preserving patient privacy. The data have been deidentified pursuant to an expert determination in accordance with the Health Insurance Portability and Accountability Act Privacy Rule. No compensation was provided to individuals whose deidentified records were included.

### Statistical Analysis

Data hosted on Nference’s nSights environment were imported on demand into the secure code workspaces deployed with Python (version 3.10.6). Missing data imputation was not undertaken. The analysis workflow uses proprietary Python packages with APIs for database querying and data standardization. The descriptive statistics were reported as n (%) and median, IQR. Loss to follow-up was considered as a censoring event for survival estimates. The Kaplan-Meier estimator from the lifelines package 0.27.7 was used in this analysis. The median rwPFS and rwOS were reported, with a 95% CI.

## Results

### Workflow Configuration

We applied 3 selection conditions to ensure that the progression captures from the clinical NLP engine are relevant and up-to-date with the patient’s current status with respect to the clinical note or report. First, entities labeled with “YES,” “PATIENT,” and “CURRENT,” each having a sentiment prediction confidence of ≥0.9, were deemed relevant. Second, to address the issue of “copy-forwarding” in clinical notes, only the first chronological instance of each extracted sentence was retained. Finally, sentences from “Past History” sections were excluded, as they are unlikely to reflect events occurring at the time of documentation. The workflow configuration is illustrated in Figure 1.

### Performance Evaluation

The accuracy of the progression capture was evaluated at 2 levels (Table 1): in level 1, sentence-level progression capture validation yielded an accuracy of 98.2% for the relevant progression captures, and in level 2, patient-level validation yielded an accuracy of 88.0%. Ablation analysis revealed the essentiality of the individual components of the clinical NLP engine for progression capture and selection conditions. All steps except the subject sentiment model labels substantially contribute to the overall model performance. This model can be disabled and the workflow performance remains the same. The patient-level workflow performance at each ablation step is outlined in Table 2.

**Table 1.** Manual validation was performed for progression at 2 levels.

		Values
**Progression capture analysis in level 1^a^ (manual validation of sampled raw progression captures [n=1000])**		
	Sensitivity	99.8%
	Specificity	96.7%
	Precision	96.6%
	Accuracy	98.2%
	*F*_1_-score	98.2
**First progression capture analysis in level 2^b^ (manual validation of first progression [n=100])**		
	Sensitivity	92.5%
	Specificity	83.0%
	Precision	86.0%
	Accuracy	88.0%
	*F*_1_-score	89.1

a Level 1: sentence-level review to validate the capture of progression sentiments at the sentence level. At this level, we reviewed the extracted sentences to ascertain the validity of the progression capture at a sentence level.

b Level 2: patient-level review to identify the first progression date. Here, we undertook a full review of patient records to ascertain the first progression capture of the metastatic disease.

**Table 2.** Output of the ablation analysis showcasing the performance metrics at each step.

	Validation against manually abstracted patient-level dataset (N=100)			
	Accuracy (%)	Sensitivity (%)	Specificity (%)	Median PFS^a^ (months), value (95% CI)
Overall workflow	88.0	92.5	83.0	20 (18‐26)
Temporal model ablation	79.0	91.5	67.9	19 (15‐23)
Subject model ablation	88.0	92.5	83.0	20 (18‐26)
Certainty model ablation	42.0	100	20.5	7 (6-8)
All 3 sentiment models ablated	35	100	15.6	6 (5-7)
Postprocessing ablation	87.0	96.2	76.6	19 (16‐23)

a PFS: progression-free survival.

### Cohort Description

The baseline characteristics are detailed in Table 3. The median age at metastasis was 59 (IQR 50.5‐69) years. The median follow-up time after metastasis diagnosis was 43.3 (IQR 28.1‐61.2) months and the median follow-up time after the start of the therapy was 39.8 (IQR 25.5‐57.9) months. The starting dose of palbociclib and letrozole was available for 53.2% and 61%, respectively. The median number of drug orders for palbociclib and letrozole was 6 (IQR 3‐12) and 4 (IQR 2‐7), respectively. The treatment characteristics, including prior and concomitant exposure to other chemotherapy agents and a history of prior radiotherapy and breast surgery, are also detailed in Table 3. The breakdown of outcomes and censoring events that contributed to the Kaplan-Meier survival estimates are further detailed in Table 4.

**Table 3.** Study cohort characteristics.

Category and variable		Overall mBC^a^ cohort (N=316)	mBC validation set (n=100)
**Demographics**			
	Age at metastasis (y), median (IQR)	59 (50.5‐68.2)	59.1 (47.8‐69.2)
	Female gender, n (%)	316 (100)	100 (100)
**Ethnicity, n (%)**			
	Not Hispanic or Latino	298 (94.3)	94 (94)
	Hispanic or Latino	11 (3.5)	3 (3)
	Unknown or choose not to disclose	7 (2.2)	3 (3)
**Race, n (%)**			
	Caucasian	293 (92.8)	95 (95)
	Asian	6 (1.8)	1 (1)
	African American or other	17 (5.4)	4 (4)
**Tumor markers, n (%)**			
	HR-positive^b^	316 (100)	100 (100)
	ER-positive^c^ and PR-positive^d^	223 (70.6)	68 (68)
	ER-positive and PR-negative	27 (8.5)	11 (11)
	PR-positive and ER-negative	50 (15.8)	15 (15)
	ER, PR status unknown	16 (5.1)	6 (6)
	HER-2/neu-negative^e^	316 (100)	100 (100)
**Disease severity, n (%)**			
	Patients with confirmed stage IV [−30,+30] within 30 d of primary diagnosis^f^	196 (61.1)	62 (62)
	ECOG^g^ performance score <3	316 (100)	100 (100)
**Disease-related follow-up, median (IQR)**			
	Follow-up after metastasis (months)	43.3 (28.1‐61.2)	50.8 (38.6‐64.2)
**Treatment, n (%)**			
	Prior systemic therapy	22 (7.5)	6 (6)
	Prior radiotherapy	125 (39.5)	38 (38)
	Prior surgical resection	128 (40.5)	37 (37)
	Other concomitant systemic therapy	36 (11)	6 (6)
**Treatment follow-up, median (IQR)**			
	Follow-up after start of treatment in months	39.8 (25.5‐57.9)	48.6 (37.8‐61.4)
	Time to treatment after advanced disease diagnosis in months	0.5 (0.2‐1.7)	0.6 (0.2‐1.7)

a mBC: metastatic breast cancer.

b HR: hormone receptor

c ER: estrogen receptor.

d PR: progesterone receptor.

e HER-2: human epidermal growth factor receptor-2.

f All patients included in the study are stage 4 cancer. The provided numbers represent those diagnosed within the stated period.

g ECOG: Eastern Cooperative Oncology Group.

**Table 4.** Breakdown analysis of outcomes and censoring events in the mBC^a^ cohort.

Source of capture			Events, n (%)
**Breakdown of progression events (n=199)**			
	**The first event is a progression capture from the pooled sources (n=152)**		
		Radiology Reports	78 (51.3)
		Clinical documents	74 (48.7)
	**The first event is the start of a second-line drug (n=47)**		
		Capecitabine	13 (27.7)
		Everolimus	11 (23.4)
		Abemaciclib	9 (19.1)
		Ribociclib	9 (19.1)
		5-Fluorouracil	3 (6.4)
		Olaparib	1 (2.1)
		Cyclophosphamide	1 (2.1)
**Breakdown of censoring events (n=117)**			
	Last encounter date		76 (65)
	Patient death date		22 (18.8)
	End of study period		19 (16.2)

a mBC: metastatic breast cancer.

### Outcomes

**Figure 3.**
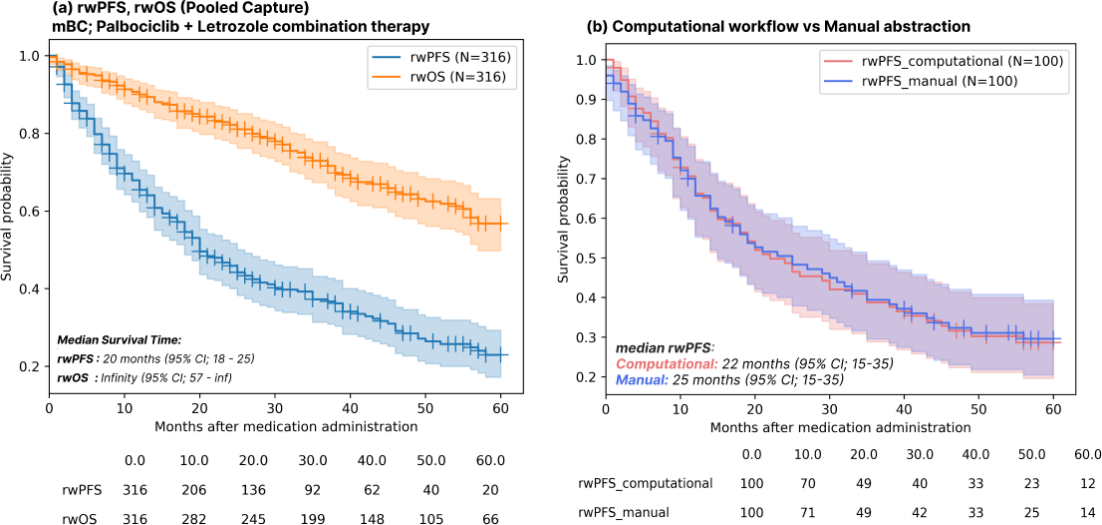
Kaplan-Meier survival plots for the overall study cohort and validation sets: (**A**) Kaplan-Meier survival plots indicating the real-world progression-free survival (rwPFS) and real-world overall survival (rwOS) in the study cohort of patients with metastatic breast cancer using pooled note sources. (**B**) Patient-level validation of first progression capture and comparing outcomes estimated by computational workflow with manual curation. mBC: metastatic breast cancer.

In the study cohort (N=316), 199 (62.9%) patients progressed during the observation period (60 mo starting from Jan 1, 2015). Out of the progressed patients, 152 (48.1%) were based on progression captures from unstructured data, and 47 (14.8%) were based on changes in the line of therapy from structured data. The median rwPFS for the overall cohort was 20 months (95% CI 18.0‐25.0; Figure 3A). The median rwOS was not reached during the study period (95% CI 57- not reached [NR]).

**Figure 4.**
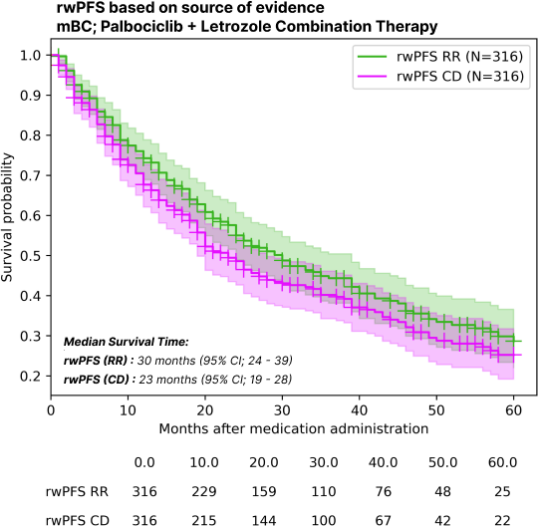
Kaplan-Meier survival plots for real-world progression-free survival (rwPFS) based on the patient note source. Survival plots indicating the real-world rwPFS with progressions captured from solitary sources of radiology reports (RR) and routine clinical documents (CD). mBC: metastatic breast cancer.

**Figure 5.**
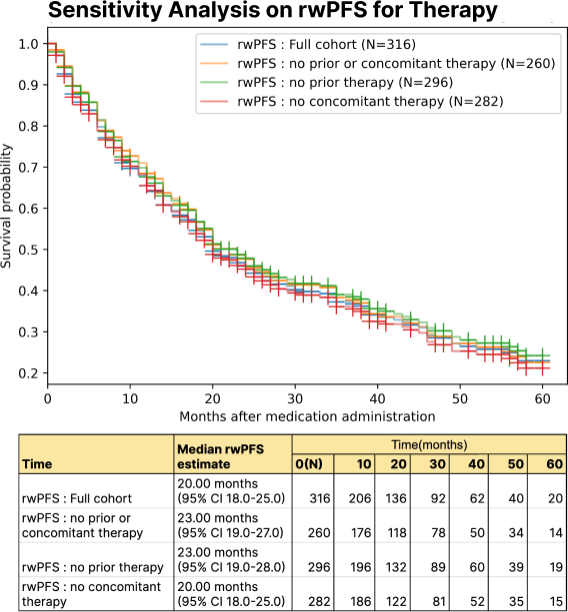
Kaplan-Meier survival curves for subgroup analysis. Each of the subgroups account for different variations in treatment patterns. The survival curves and risks table showcase the effect of other prior or concomitant systemic therapies on the median real-world progression-free survival (rwPFS).

In the mBC validation set of 100 patients, the median rwPFS was determined to be 25 (95% CI 15-35) months by manual curation and 22 (95%CI 15-35) months by the computational workflow outlined in Figure 3B. Based on the data source for progression capture, the rwPFS estimated exclusively from radiology reports was 30 (95% CI 24.0-39.0) months, compared to 23 (95% CI 19.0-28.0) months when estimated exclusively from CDs, as represented in Figure 4. Subgroup analysis on the rwPFS based on prior or concomitant therapies is detailed in Figure 5.

### External Dataset Validation

External validation was conducted using data from a partner AMC, representing a health system distinct from the main study, to assess the generalizability and robustness of the progression capture pipeline. At Level 1, manual validation of 200 sampled raw progression captures achieved an accuracy of 92.5% and an *F*_1_-score of 92.8%, while Level 2 validation of the first progression in 61 patients reported an accuracy of 90.2% and an *F*_1_-score of 92.5%. Two patients were excluded from performance metrics because the textual evidence identified during manual abstraction of the first progression event was unavailable to the automated extraction pipeline. Comprehensive performance metrics, including sensitivity, specificity, and *F*_1_-scores, are detailed in Table 5.

**Table 5.** Manual validation of the progression capture workflow on the external dataset.

Validation step	Sensitivity	Specificity	Accuracy	Precision	*F*_1_-score
**Level 1**					
Manual validation of sampled raw progression captures (n=200)	89.7%	95.7%	92.5%	96%	92.8%
**Level 2**					
Manual validation of first progression (n=61)	97.4%	78.3%	90.2%	88.1%	92.5%

### Sensitivity Analysis on rwPFS Estimates

The sensitivity analysis demonstrated that rwPFS estimates were robust under varying conditions. Systematic removal of 10%, 20%, and 30% of progression events resulted in median rwPFS values of 20 (95% CI 18-26) months, 20 (95% CI 18-27) months, 22 (95% CI 18-28) months, and 23 (95% CI 19-29) months for the complete, 10%, 20%, and 30% datasets, respectively, with widening CIs indicating increased uncertainty. Similarly, median rwPFS estimates were comparable when death was treated as censorship versus a progression event, with values of 20 months (95% CI 18-25) and 18 months (95% CI 15-21) in the main dataset. These findings, along with overlapping CIs, indicate that the rwPFS estimates were not meaningfully affected by missing data or event definitions. See Figure S5 in Multimedia Appendix 1 for Kaplan-Meier curves and event tables.

### PHQ-8 Outcomes and Disease Progression

Of the 316 patients, 94 had at least 2 PHQ-8 scores recorded during the study period, including 30 nonprogressed and 64 progressed patients. Nonprogressed patients showed greater mean peak reduction (5.57, SD 5.90 vs 2.95, SD 4.30; *t*_92_=−2.397, *P*=.02) and cumulative decline (mean 8.00, SD 12.68 vs 3.66, SD 6.40; *t*_92_=−2.201, *P*=.03) in PHQ-8 scores compared to progressed patients. See Table S3 in Multimedia Appendix 1 for details.

## Discussion

### Overview

The study showcases the development and validation of a novel semiautomated workflow for estimating rwPFS in patients with mBC using deidentified EHRs. One of its key strengths lies in the integration of NLP techniques to extract clinician-reported progression events from unstructured data sources such as clinical notes and radiology reports, combined with structured patient data like drug orders and clinical encounters. This approach enhances the accuracy and comprehensiveness of capturing progression events, as evidenced by the high sensitivity (99.8%) and specificity (96.7%) at the sentence level with good patient-level accuracy (88%).

While our initial goal was to develop a fully automated workflow for capturing disease progression, we have successfully implemented a semiautomated approach. This is advantageous because the semiautomated method allows for disease-specific adjustments to clinical concept recognition configurations, ensuring relevance across various cancer types. This flexibility underscores the potential for broader applicability beyond mBC, making it a valuable tool for oncological research and RWE generation.

### Principal Findings

The median rwPFS of 20 months (95% CI 18‐25) reported in this study is comparable to those reported in previous real-world studies and clinical trial results, validating the workflow’s reliability and accuracy [20,21]. Subgroup analysis revealed the impact of prior and other concomitant medications on median rwPFS. For instance, the PALOMA-2 trial reported a median progression-free survival (PFS) of 24.8 months (95% CI 22.1-inf), which is comparable to the real-world observation in the study subcohort (N=260) of patients who received palbociclib and letrozole in the first-line metastatic setting (patients with no other prior or concomitant drugs), which was 23.00 months [22]. A matched comparison between the study cohort and other real-world cohorts or clinical trials could further establish the concordance between the survival estimates. Integrating progression events from both radiology reports and routine clinical or oncology notes standardizes the identification of disease progression, mitigating biases and overestimation that can arise from relying exclusively on a single data source. Ablation analysis also revealed the futility of using the subject sentiment analysis model in the workflow, as physicians are unlikely to describe the progression status of a family member or a blood relative in the patient’s notes. While this model is useful for extracting other concepts using the clinical NLP engine, it has shown no benefit in its usage for progression capture.

The external validation further demonstrated the robustness and generalizability of the progression capture workflow across health systems. Manual validation on this dataset also achieves high accuracy at the sentence level (92.5%) and at capturing the first progression event (90.2%). Sensitivity analysis confirmed that rwPFS estimates were stable, regardless of whether death was treated as censorship or an event, with overlapping CIs observed across both scenarios. Sensitivity analysis confirmed that rwPFS estimates were stable across varying levels of missing data and event definitions, with slight increases in median rwPFS and wider CIs under data incompleteness and overlapping intervals when treating death as censorship or an event. Furthermore, integration of PHQ-8 outcomes revealed significant associations between patient-reported mental health and progression status, highlighting the potential of PROs to provide complementary insights.

### Comparison to Prior Work

The study also highlights the importance of source data used for determining rwPFS. Relying solely on radiology reports overestimated the median rwPFS compared to estimates derived from both clinical notes and radiology reports combined. The median rwPFS from the pool of free-text data excluding radiology reports (23 months) was closer to the median survival of the overall study cohort with all available pooled free-text data (20 months) when compared to the median rwPFS computed from free-text data exclusively from radiology reports (30 months). This discrepancy can be explained by the observation that patients can undergo radiological evaluations outside the EHR data network, with their findings being documented by treating physicians within the EHR network in patients’ routine clinical notes. Similar findings were observed in a previous study that analyzed the impact of source data on real-world survival estimates [6]. Additionally, relying solely on structured data like drug records (time to discontinuation or time to next treatment) as a surrogate for rwPFS has been shown to underestimate the median rwPFS substantially in a prior study [23]. PROs provide direct insight into a patient’s symptoms and quality of life and have been linked to progression-free and overall survival in prior studies. Although direct comparisons with alternative workflows were not performed, our method demonstrates performance metrics that are in line with those reported in previous studies, warranting further comparative analyses in future work.

Among other computational techniques for characterizing cancer response in real-world data (RWD), the use of LLMs has also shown promise. A prior study evaluating this has shown GatorTron to be the best-performing model, achieving an accuracy of 89% at the radiology report level upon fine-tuning [24]. However, applying LLMs across a broader patient corpus needs further investigation to fully ascertain their validity and generalizability. PROs provide direct insight into a patient’s symptoms and quality of life and have been linked to progression-free and overall survival in prior studies [25-27]. We have observed significant associations between the decline in PHQ-8 scores and the patient’s progression status.

### Limitations

There are, however, limitations to this study. First, the reliance on clinician-reported events means that the accuracy of the workflow is reliant on the quality and completeness of clinical documentation. Incomplete or inconsistent documentation could lead to underestimation or overestimation of progression events. To mitigate this, careful validation of extraction patterns and data completeness checks were implemented. Second, although the semiautomated workflow reduces the resource-intensive nature of manual abstraction, it requires initial manual rule definition and configuration, which could introduce biases based on the selected rules and criteria. Representative evaluation samples were curated across the breast cancer cohorts to reduce the biases. Third, sensitivity analyses were limited primarily to variations in clinical text sources and censoring definitions. Expanding sensitivity analyses to include demographic factors, alternative definitions of progression, and data-source reliability could further strengthen the robustness of the findings. Fourth, augmenting the mortality data with commercial and federal death registries could enhance the accuracy of survival estimates. This was not feasible in the present analysis but represents an important area for future improvement. Fifth, integration of PROs could provide a more comprehensive understanding of patient well-being in relation to progression events. However, demonstrating this in RWD was challenging due to the limited availability of patient-reported records. Finally, the ensemble deep learning engine’s performance was evaluated within a specific cohort of mBC patients; thus, further validation across more diverse external datasets and different cancer types is necessary to truly establish the generalizability of the workflow.

### Future Directions

These findings align with the growing body of research advocating for integrating artificial intelligence and machine learning in health care data analysis, as these technologies can substantially enhance the speed, accuracy, and breadth of data processing capabilities. Future work will explore more advanced text data analysis and extraction methods, such as adaptive machine learning techniques and LLMs, to minimize manual import and enhance scalability. Furthermore, by using federated learning, insights and patterns from diverse populations across various institutions can be pooled securely, enriching the model’s generalizability and performance across different health care settings. The successful implementation of this automated workflow demonstrates its potential to streamline the data extraction process from EHRs from various health systems. It also paves the way for its application in other oncological studies, where similar challenges in data abstraction exist.

### Conclusions

Developing a practical and scalable method for capturing real-world progression from EHR data is crucial to improving oncological research and patient care. Overall, this technology represents a step forward in realizing the full potential of EHR data in oncology. Our findings establish a workflow for automated data capture to provide a more efficient and scalable method than traditional manual processes, particularly in handling complex, unstructured EHR data. Although the principles of progression capture remain the same across other cancer types, further research across other types of solid tumors is needed to ascertain the generalizability of the workflow.

## Supplementary material

10.2196/64697 Multimedia Appendix 1 Additional tables and figures.

## Abbreviations

AMC: academic medical center
API: application programming interface
BERT: Bidirectional Encoder Representations from Transformers
CD: clinical document
EHR: electronic health record
HER-2: human epidermal growth factor receptor-2
*ICD-10*: *International Statistical Classification of Diseases, Tenth Revision*
*ICD-9*: *International Classification of Diseases, Ninth Revision*
LLM: large language model
mBC: metastatic breast cancer
NLP: natural language processing
PFS: progression-free survival
PHI: protected health information
PHQ-8: Patient Health Questionnaire-8
PRO: patient-reported outcome
RECIST: response evaluation criteria in solid tumors
RWD: real-world data
RWE: real-world evidence
rwOS: real-world overall survival
rwP: real-world progression
rwPFS: real-world progression-free survival
