# Supplementary material for: A Deep Learning–Enabled Workflow to Estimate Real-World Progression-Free Survival in Patients With Metastatic Breast Cancer: Study Using Deidentified Electronic Health Records
Source: JMIR Cancer. 2025 May 15;11:e64697. doi: 10.2196/64697 (PMC12097284; doi:10.2196/64697)
Supplement: Multimedia Appendix 1 [file cancer-v11-e64697-s001.docx]

### SUPPLEMENTARY ITEMS

#### Supplementary Note 1. Clinical NLP engine:

Clinical NLP engine is an assortment of 5 Deep Neural Network Models trained to extract relevant information from clinical notes. Patient notes contain a lot of vital information useful in making necessary predictions. However, since they are present in an unstructured format, i.e., sentences and notes, we cannot use them directly. Clinical NLP engine was developed to accurately extract relevant information from the notes and translate unstructured patient data into structured data elements. The models were initially trained on a corpus of double-tagged 75k human-annotated sentences extracted from de-identified free text data of the AMC network available in the nference nSights platform. The models are further improved using annotated datasets generated by fine-tuned large language model(LLM) agents.

For named entity recognition, the model was trained to recognize clinical concepts such as problems, medications, labels using masked token prediction. For association tasks, where relationships (e.g., disease-severity or drug disease) between entities are inferred, the engine uses masked modeling on entity pairs. The maximum sequence length for these models is 128 tokens to reduce computational overhead and enable efficient inference. Experimental evaluations during development demonstrated that masked modeling outperformed other techniques by effectively narrowing the model’s context without expanding the input sequence length.

The information can be divided into two aspects:

1. Entities: All the medically relevant nouns and adjectives present in notes. It is divided into three categories:
   1. Primary Entities: Entities with a proper definition (mostly nouns) like PROBLEM, MEDICINE, DIAGNOSTIC_PROCEDURE…
   2. Secondary Entities: Entities that advise on or provide value to a primary entity. Like VALUE, MED_DOSE, SEVERITY…
   3. Associations: A combination of 2 entities that are associated with each other.
2. Sentiments: Sentiments are the context a sentence indicates towards the entity/entities present. It can be very subjective. We focus on 3 different event-based aspects.
   1. Temporal Assessment: Describes the timeline associated with the entity of interest with respect to the sentence
   2. Certainty Assessment: Describes if the entity or an event certainly transpired, given the context of the sentence.
   3. Subject Assessment: Describes whether the entity in the sentence is associated with the patient.

Clinical NLP engine consists of an assortment of the following 5 Deep Neural Network Models:

1. Clinical Named Entity Recognition
2. Certainty Assessment Model
3. Temporality Assessment Model
4. Subject Classification Model
5. Association Model

### SUPPLEMENTARY TABLES

| **Supplementary Table 1.** Technical specifications of the model Healthcare API and search patterns used for progression capture. | | |
| --- | --- | --- |
| Pattern description | | regex pattern |
| new [0-5 words] lesion/tumor/mass/growth | | (?i)(new)((( )([a-z]*)){0,5})( )(lesions?\|tumou?rs?\|mass((es)?)\|growth\|disease)   |
| lesion/tumor/mass/growth/disease [0-5 words] progression/sive | | (?i)(lesions?\|tumou?rs?\|mass((es)?)\|growth\|disease)((( )([a-z]*)){0,5})( )(progress[a-z]+)   |
| progression/sive [0-5 words] lesion/tumor/mass/growth/disease | | (?i)(progress[a-z]+)((( )([a-z]*)){0,5})( )(lesions?\|tumou?rs?\|mass((es)?)\|growth\|disease)   |

#### Validation Metrics of Clinical NLP Engine:

**Supplementary Table 2.** Model specific performance metrics across different tasks.

| **Label** | **F1 Score** | **Support** |
| --- | --- | --- |
| NER Model |  |  |
| PROBLEM | 0.85 | 5936 |
| LAB_DATA | 0.91 | 2127 |
| DIAGNOSTIC_PROCEDURE | 0.84 | 781 |
| MEDICINE | 0.91 | 2414 |
| ANATOMICAL_STRUCTURE | 0.81 | 2324 |
| BODY_MEASUREMENT | 0.86 | 1624 |
| RESULT | 0.78 | 757 |
| SEVERITY | 0.80 | 534 |
| UNIT | 0.90 | 1298 |
| VALUE | 0.92 | 3350 |
| Certainty Model |  |  |
| YES | 0.96 | 4503 |
| Temporality Model |  |  |
| CURRENT | 0.86 | 3480 |
| Subject Model |  |  |
| PATIENT | 0.98 | 4758 |
| Association Model |  |  |
| LAB_DATA -- VALUE | 0.99 | 21265 |
| UNIT -- VALUE | 0.99 | 6334 |
| LAB_DATA -- RESULT | 0.99 | 3054 |
| ANATOMICAL_STRUCTURE -- PROBLEM | 0.97 | 1304 |
| BODY_MEASUREMENT -- VALUE | 1.00 | 1112 |
| BODY_MEASUREMENT -- RESULT | 0.96 | 898 |
| PROBLEM -- SEVERITY | 0.96 | 837 |
| ANATOMICAL_STRUCTURE -- BODY_MEASUREMENT | 0.97 | 299 |
| DIAGNOSTIC_PROCEDURE -- PROBLEM | 0.89 | 250 |
| LAB_DATA -- PROBLEM | 0.78 | 174 |
| ANATOMICAL_STRUCTURE -- DIAGNOSTIC_PROCEDURE | 0.98 | 131 |
| DIAGNOSTIC_PROCEDURE -- RESULT | 0.95 | 96 |
| The metrics above demonstrate the performance of the Nference Clinical NLP Engine. The test dataset was based on a manually tagged gold standard dataset. **Support** represents the sample size of the test dataset, and the **label** represents a selective list of labels within the Nference Clinical NLP Engine utilized in the study. The overall experimental results are unpublished and will be published separately. | | |

| **Supplementary Table 3.** PHQ-8 Outcomes and Disease Progression | | | | |
| --- | --- | --- | --- | --- |
| **Metric** | **Non-Progressed (n=30)** | **Progressed (n=64)** | **Test Statistic** | **p-value** |
| Peak Reduction  (Mean ± SD) | 5.57 ± 5.90 | 2.95 ± 4.30 | t = -2.397 | 0.018 |
| Cumulative Decline  (Mean ± SD) | 8.00 ± 12.68 | 3.66 ± 6.40 | t = -2.201 | 0.03 |

### SUPPLEMENTARY FIGURES


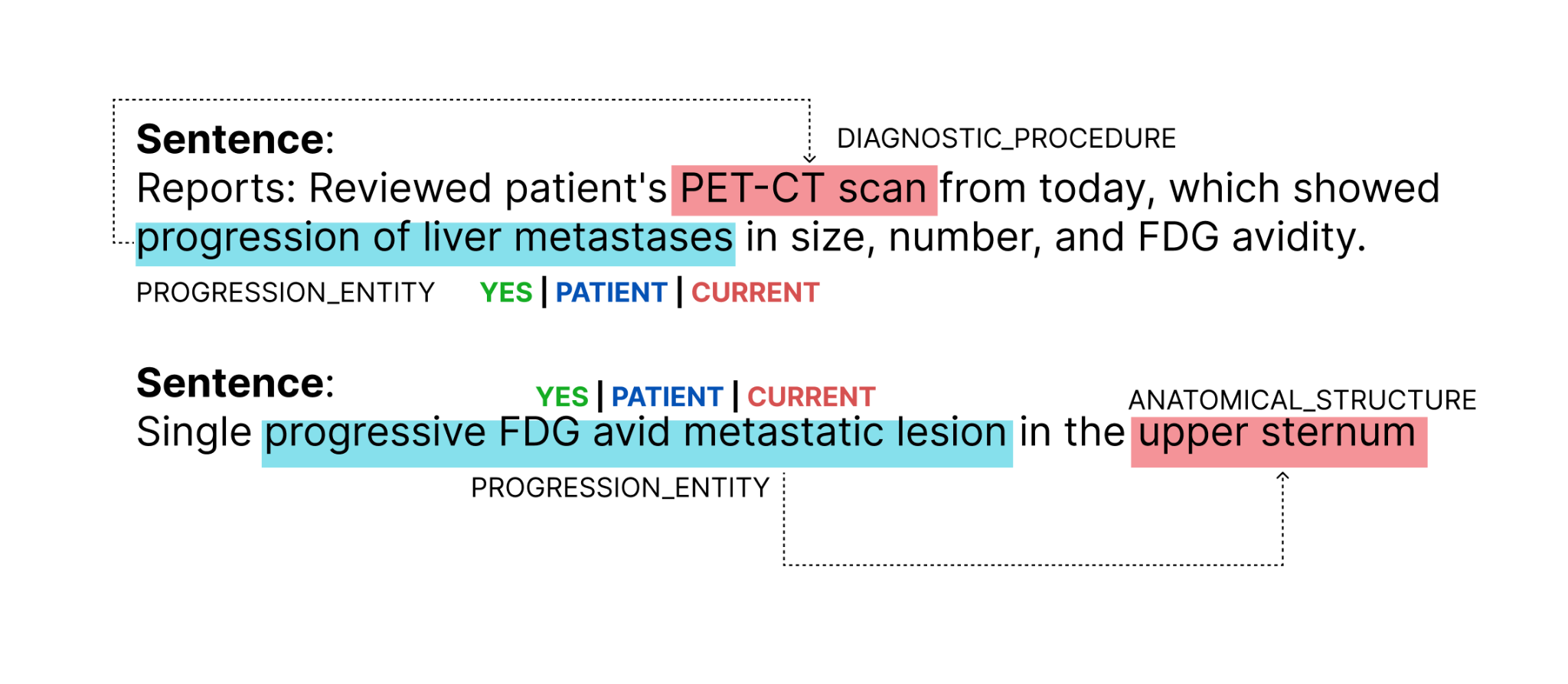


**Supplementary Figure 1.** **Entity Recognition and Association by Clinical NLP Engine.** The figure illustrates how the clinical NLP engine identifies, labels, and links the entities in a sample tokenized sentence.

**
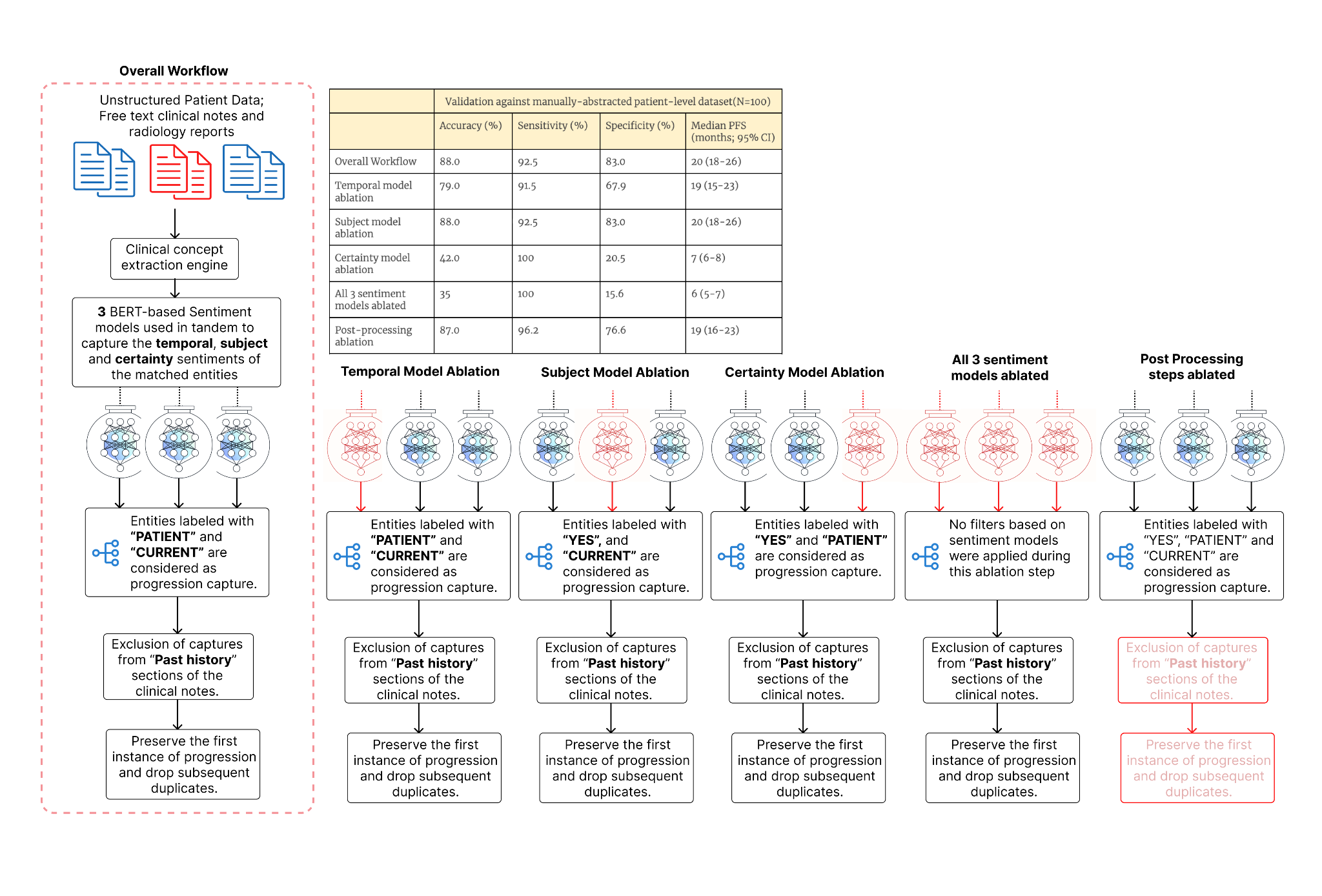
Supplementary Figure 2. Ablation Analysis.** Illustrates the ablation steps and highlights components that were ablated at each step.

**
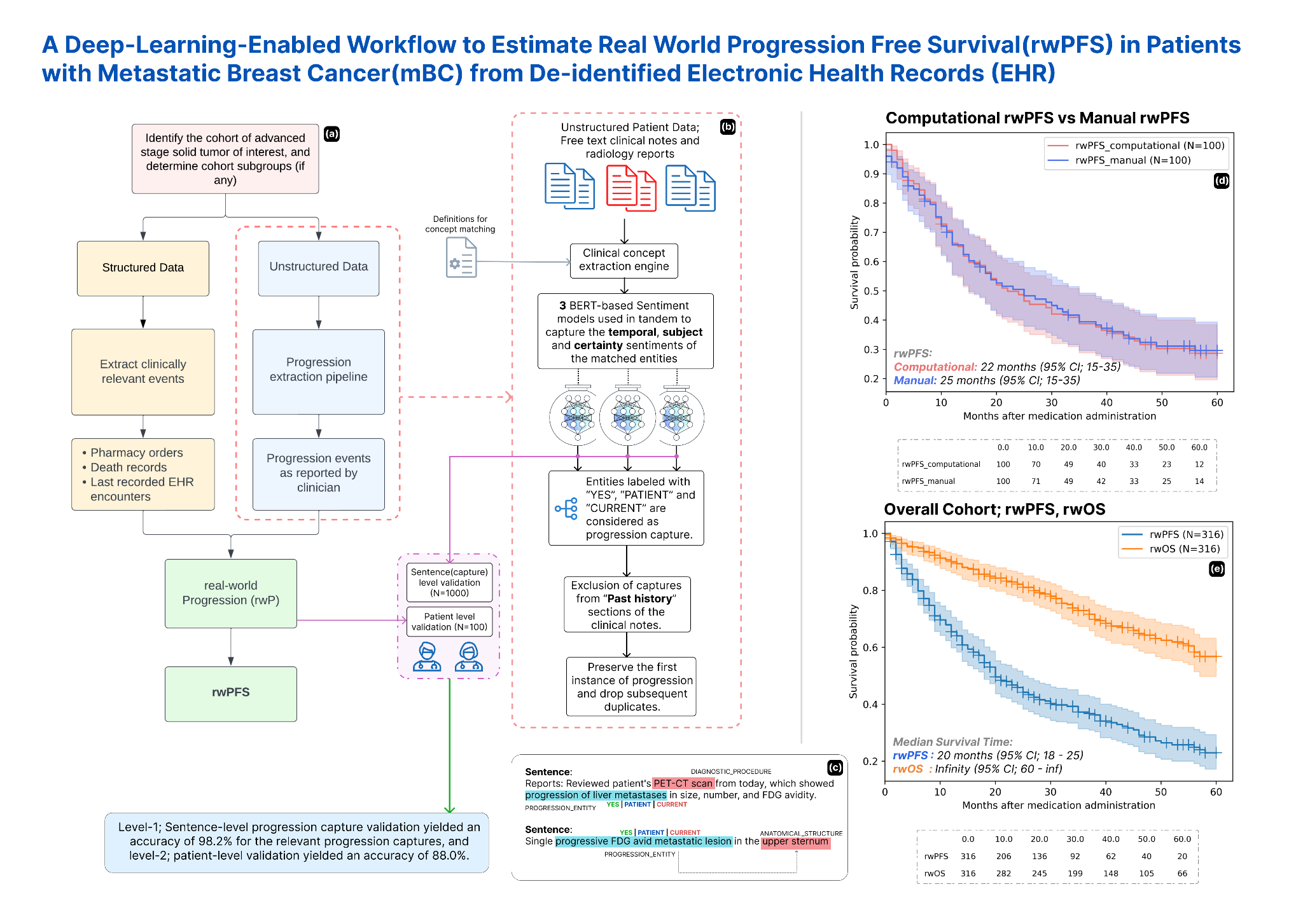
Supplementary Figure 3. High-level Workflow Overview** (a) Illustrates the high-level overview of the workflow for real-world progression(rwP) extraction and estimating rwPFS. (b) Components of the clinical NLP engine for progression extraction from free-text clinical documents and radiology reports. (c) Demonstrates how the clinical NLP engine identifies, labels, and links entities. (d, e) Kaplan-Meier curves for rwPFS estimates by computation and manual workflows of palbociclib and letrozole combination therapy in mBC validation set(N=100) and the overall cohort(N=316)

**
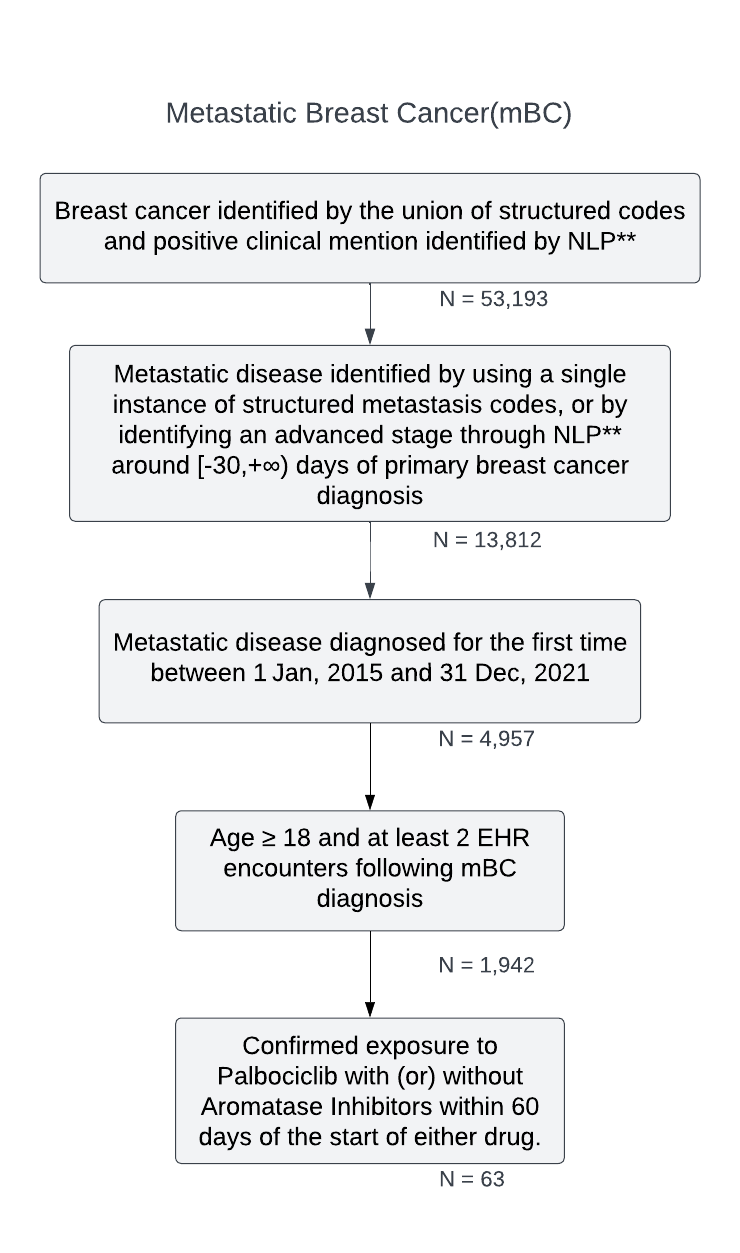
**

**Supplementary Figure 4.**  **Cohort Attrition of patients in the External validation Dataset.** Structured codes 174*(ICD-9), and C50* (ICD-10) or >4 positive disease sentiments from the augmented curation disease diagnosis model were used for breast cancer. For evidence of metastasis 197*, 198* (ICD-9), C78*, and C79* (ICD-10) in conjunction with augmented curation was used; *(single asterisk) represents all the children codes within the parent code. **(double asterisk) NLP - Natural Language Processing.

**
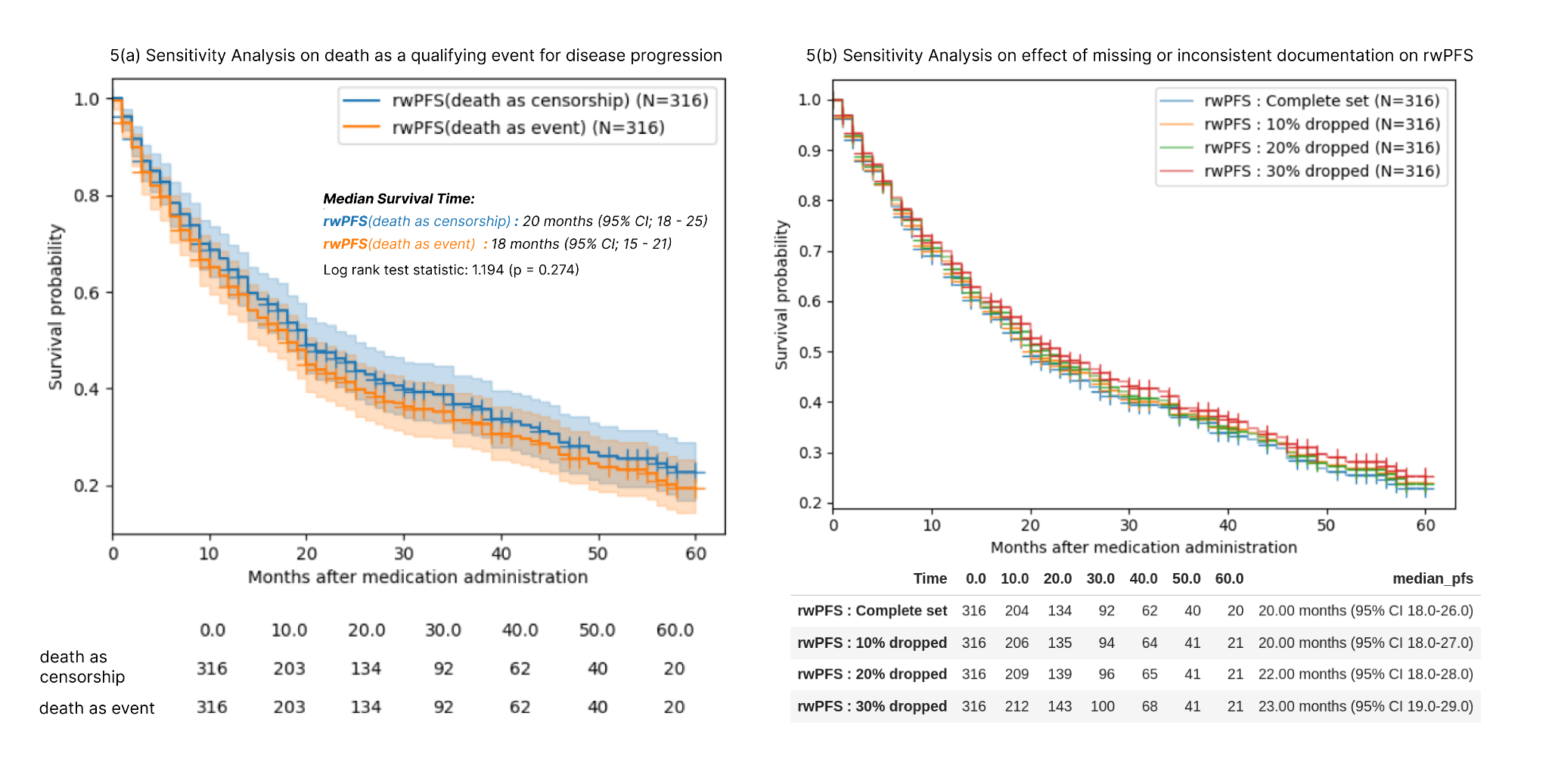
**

**Supplementary Figure 5. Sensitivity Analysis** to assess the robustness of real-world progression-free survival estimates (a) Kaplan-Meier Survival plots indicating the effect of death as a qualifying event for progression. (b) Kaplan-Meier Survival plots indicating the effect of missing source data, due to inconsistent documentation of progression, representing Complete set, 10%,20%, 30% missingness at random.
